# ZnIn_2_Se_4_ nanoparticles photocatalyst for efficient solar fuel production

**DOI:** 10.1016/j.isci.2024.110422

**Published:** 2024-06-29

**Authors:** Yinyin Ai, Yukun Li, Ting Li, Ruohan Hou, Qing Wang, Aneela Habib, Guosheng Shao, Peng Zhang

**Affiliations:** 1 State Center for International Cooperation on Designer Low-carbon and Environmental Materials (CDLCEM), School of Materials Science and Engineering, Zhengzhou University, Zhengzhou 450001, China

**Keywords:** Catalysis, Energy engineering, Nanomaterials

## Abstract

Selecting a suitable photocatalyst to establish the Z-scheme heterojunction which is accompanied by effective photogenerated hole and electron separation, is one of the advantageous strategies for efficient photocatalytic solar energy conversion. Therefore, we prepared a ZnIn_2_Se_4_ nanoparticles photocatalyst to build a double Z-scheme heterojunction with mixed-phase TiO_2_ nanofibers, boosting photocatalytic solar fuel preparation. The result of X-ray photoelectron spectroscopy confirmed the existence of interfacial chemical bonds and internal electric fields. The interfacial Ti-Se bond is regarded as a channel and the internal electric field serves as the driving force for electron transfer. And the composite photocatalyst exhibits a great hydrogen evolution rate of 0.11 mmol g^−1^ h^−1^. From a forward-working perspective, this work proposes a ZnIn_2_Se_4_ nanoparticles photocatalyst for efficient solar fuel conversion, promoting the application of bimetallic selenide photocatalyst in the field of photocatalysis.

## Highlights

• ZnIn_2_Se_4_ nanoparticles photocatalyst was utilized for solar fuel production
• Homojunction TiO_2_ improved the efficiency of photogenerated carrier separation
• Internal electric field and interfacial chemical bond were constructed

## Graphical abstract

**Figure:**
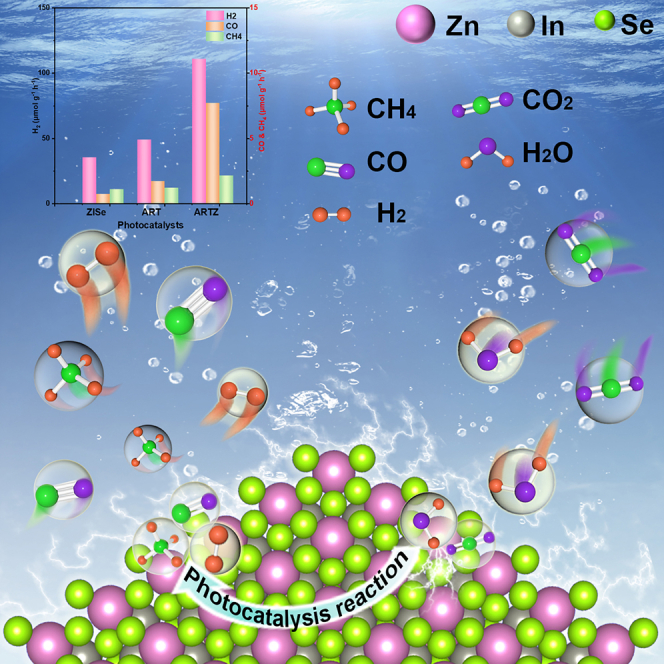

## Introduction

The ever-rapid development of industrialized societies has led to a drastic consumption of fossil fuels, which has caused intolerable energy and environmental issues.^1^^,^^2^^,^^3^ The existence of these problems has guided the exploration of green and renewable energy to deal with.^4^^,^^5^^,^^6^ Solar energy has gained significant attention from the scientific research community due to its abundant reserves and accessibility, making it a popular choice among lots of clean energy.^7^^,^^8^^,^^9^^,^^10^ Over the years, researchers have utilized the solar energy conversion to produce green and high-energy fuels inspired by photosynthesis in nature, such as hydrogen, methanol, methane, and carbon monoxide.^11^^,^^12^^,^^13^ The unimaginably weak efficiency of simple solar energy conversion without the intervention of additional condition, however, researchers come up with photocatalytic reactions to efficiently achieve.^14^^,^^15^^,^^16^ Photocatalyst, as the core of photocatalytic reaction, therefore, the selection of outstanding photocatalyst is the key to promote photocatalytic solar energy conversion rate.^17^^,^^18^

Bimetal-halide semiconductor photocatalysts, due to their charming photoelectric performance and efficient capture of sunlight, have been attracted large-scale interest.^19^^,^^20^^,^^21^ Bimetal-selenide ZnIn_2_Se_4_, as a member of defective chalcopyrite, is devoted extensively to solar cells and optoelectronic devices. Moreover, the Se atom of ZnIn_2_Se_4_ has a larger atomic radius which is weak to bind outer shell electrons compared with the traditional ZnIn_2_S_4_ photocatalyst.^22^^,^^23^^,^^24^^,^^25^ As a kind of semiconductor, the conduction band of ZnIn_2_Se_4_ is in a more negative position, indicating that it has a stronger photocatalytic reduction ability.^26^^,^^27^ Therefore, this bimetal-selenide, serves as a semiconductor photocatalyst, can improve solar energy conversion to a great extent. The preparation of ZnIn_2_Se_4_ mainly focuses on Vertical Bridgman and chemical vapor deposition, which are all with technical challenges.^28^^,^^29^ Guo et al. developed a solvothermal method synthesis of ZnIn_2_Se_4_ semiconductor and applied it in the field of photocatalysis. Inspired by this, the photocatalyst can be synthesized by a simple and fast solvothermal method.^27^ These results show that choosing a simple one-step solvothermal method to synthesize this rare and scarce semiconductor ZnIn_2_Se_4_ as a photocatalyst is convenient in the study of photocatalytic solar energy production process.

Nevertheless, the utilization of ZnIn_2_Se_4_ semiconductors in photocatalysis is constrained by several limitations, including the formation of substantial agglomerates, the facile recombination of photogenerated carriers, and the relatively low valence band potentials. Consequently, the photocatalytic efficacy of ZnIn_2_Se_4_ is suboptimal. The selection of appropriate loading templates for the construction of ZnIn_2_Se_4_ heterojunctions can effectively enhance the dispersion of particles, augment the number of active sites, inhibit the recombination of photogenerated carriers, and facilitate the redox reaction, thereby representing an efficacious strategy to enhance the performance of ZnIn_2_Se_4_ in the preparation of solar fuels.^30^^,^^31^ The accompanying result is that the quantity of active sites will decrease, which in turn reduces its photocatalytic performance. Semiconductor nanofibers, such as BiVO_4_, SrTiO_3_, BaTiO_3_, TiO_2_, and ZnO nanofibers, with the charming length-diameter ratio is used as the loading template to effectively avoid the problem of ZnIn_2_Se_4_ (ZISe) nanoparticles agglomeration.^32^^,^^33^^,^^34^^,^^35^^,^^36^^,^^37^ TiO_2_ NFs, as traditional semiconductor nanofibers photocatalysts, are regarded as the primary alternative photocatalytic material owing to their inexpensive preparation cost, high physicochemical stability, and easy synthetic technology.^38^^,^^39^^,^^40^ Regrettably, there are two big issues in this photocatalyst, wide bandgap and rapid recombination of photogenerated electron-hole pairs, which decrease photocatalytic efficiency. In the review of research findings, constructing anatase TiO_2_ (AT) and rutile TiO_2_ (RT) homojunction (ART) could significantly promote the charge transformation at the surface.^41^^,^^42^ The Presence of the heterophase homojunction will tremendously improve the separation and migration of photogenerated carriers. In addition, heterojunction constructed by coupling ZISe NPs and RT NFs creates an interfacial Ti-Se bond for a convenient electronic transmission channel to further boost photocatalytic hydrogen evolution and carbon dioxide reduction.^42^^,^^43^^,^^44^ Concurrently, the modulation of internal electric fields appears as a provider of driving force, resulting in a fast transfer rate of charge at the interface position after these substances form a close connection.^45^^,^^46^^,^^47^

In this work, we prepared ART homojunction NFs and ZISe NPs (ARTZ) combined with electrospinning technology and simple solvothermal method, through interfacial Ti-Se bond and internal electric field constructed double Z-scheme heterojunction. On the one hand, the ZISe NPs with excellent photoelectric performance and great sunlight absorption, improved the utilization efficiency of sunlight-excited carriers in the system.^16^^,^^31^ On the other hand, the homojunction of ART NFs not only was optimized to inhibit the recombination of self-internal carriers, but also provided with a load template and carriers separator for ZISe NPs.^38^^,^^48^^,^^49^ With the formation of interfacial Ti-Se bonds, the photogenerated electrons were equivalent to possess a more convenient transmission channel. Besides, the mechanism of double Z-scheme charge transfer has been proved by ISI-XPS, and electron transport abided by the track transmission mode from AT to RT to ZISe. The double Z-scheme heterojunction admirable photocatalytic solar energy evolution rate and great electrochemical stability. Utilization of ZISe NPs semiconductor has created more understanding of bimetal-halide semiconductor in the academic community and is expected to obtain a higher level in the future photoelectric area.

## Results and discussion

### Crystalline phase and morphology characterization

**Figure 1:**
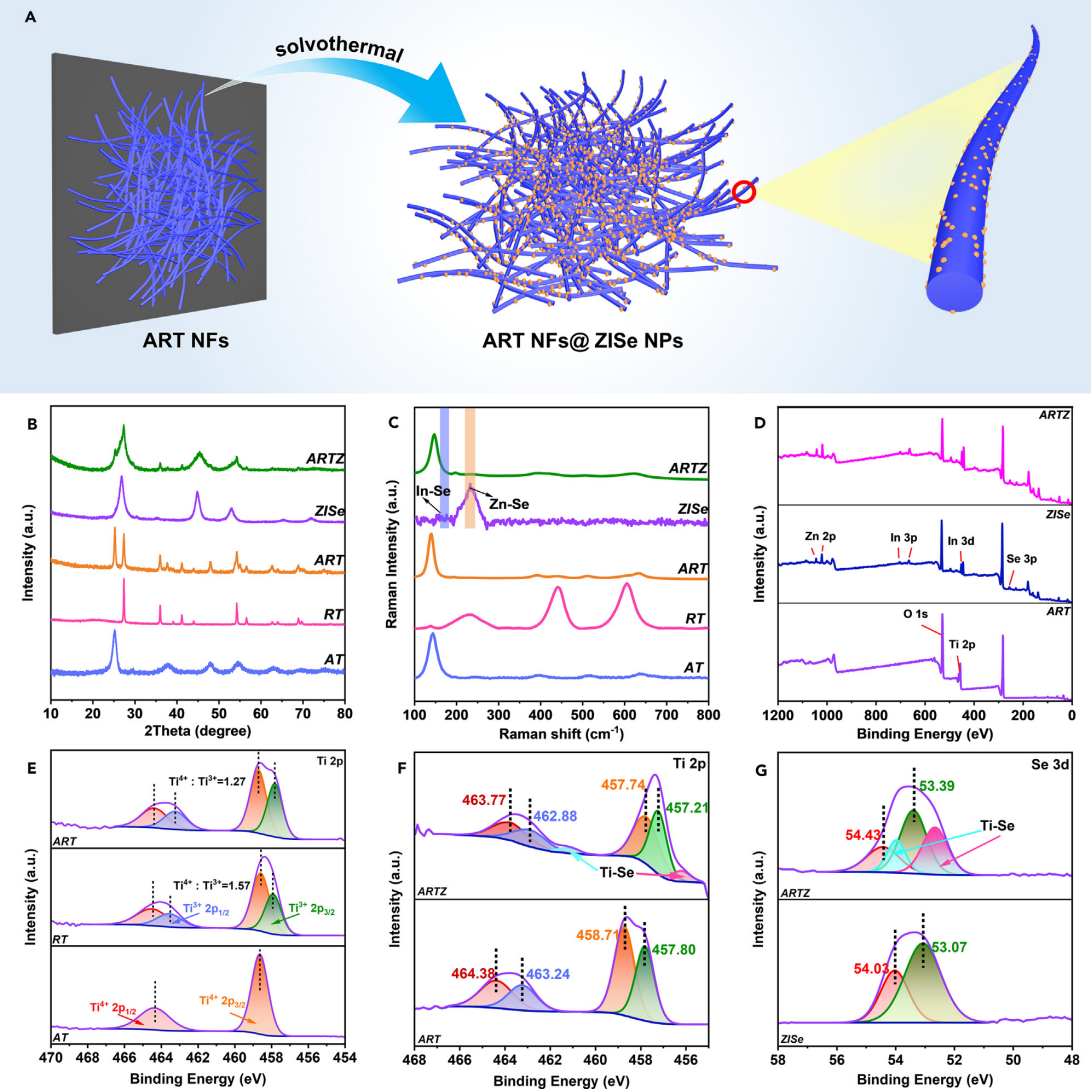
The preparation and crystalline of samples (A) the synthesis process of ARTZ photocatalyst. (B) XRD patterns. (C) Raman patterns of AT, RT, ART, ZISe and ARTZ. (D) The XPS spectra of ZISe, ART and ARTZ. (E) The Ti 2p of AT, RT and ART. (F) The Ti 2p of ART and ARTZ. (G) The Se 3days of ZISe and ARTZ.

The preparation of composite ARTZ is exhibited as shown in Figure 1A. One-dimensional nanofibers were obtained using electrospinning technology, with more detailed preparation steps provided in Figure S1A and method details. By regulating different temperatures of preoxidation treatment during this process to 450, 600, and 800°C, respectively, AT, ART, and RT NFs could be obtained. The different nanofibers were prepared to deal with the solvothermal method at 180°C for 12 h. During the solvothermal synthesis reaction, the ZISe NPs *in-situ* scattered growth on the nanofibers while serving as the load template. Figure 1B demonstrates the X-ray diffraction (XRD) patterns of AT (JCPDS: 21–1272), RT (JCPDS: 21–1276), ZISe (JCPDS: 39–0458) and ARTZ, and the crystal structure of three semiconductors are shown in Figure S1B. Regarding TiO_2_ nanofibers, the crystal phases gradually transitioned from a single anatase phase to mixed-phase anatase and rutile, and finally to a single rutile phase as the temperature increased from 450°C to 800°C. Additionally, the XRD pattern of ZISe was a good match. The bimetallic selenide photocatalyst demonstrated the unambiguous peaks at 26.8°, 44.9°, 53.1° and 72.1°, which correspond to the (112), (204), (312) and (316) crystal planes. Furthermore, the XRD patterns of ARTZ exhibited three simultaneous categories of peaks, confirming the successful synthesis of the ARTZ photocatalyst. The vibrating peaks of the target photocatalyst were further measured by Raman spectroscopy in Figure 1C. For ZISe NPs, there were vibrations of In-Se and Zn-Se bonds vibration at the 166 and 233 cm^−1^, respectively. The strong signal of ART vibrating peaks masked the peaks of ZISe NPs in the composite. These all results confirm the perfect synthesis of ART NFs, ZISe NPs, and ARTZ composite photocatalyst.

The X-ray, with energy higher than the binding energy of the nucleus, was interacted with the surface atoms of the sample, exciting electrons and causing them to break away and become free electrons.^50^^,^^51^^,^^52^^,^^53^ The X-ray photoelectron spectroscopy (XPS) of ART, ZISe, and ARTZ photocatalysts testified that the peaks of relevant elements all appeared and there was no incidental element in Figure 1D, which is consistent with element mapping. Calcination at high temperatures (650°C–800°C) could lead to oxygen vacancies (OVs) due to insufficient oxygen. The XPS Ti 2p spectrum (Figure 1E) showed the vibrational peaks of Ti^3+^. The XPS spectrum of Ti 2p and Se 3days, shown in Figures 1F and 1G, revealed that the peaks of interfacial chemical Ti-Se bonds were at 456.21, 461.27 eV and 52.65, 53.95 eV.^54^^,^^55^ As found from the Figures S2A and S2B), the spectrum peaks of Zn 2p and In 3 days for ARTZ composite exhibited positive shift compared with original ZISe, resulting in the enriched positive charge at the interface near the ZISe side, while the negative charge concentrating near the RT side accordingly. As a consequence, the internal electric field was formed from ZISe to RT. These interfacial chemical bonds acted as a bridge for electron transport, providing the wonderful conditions for rapid charge transfer between heterojunctions. All the above results demonstrate that the composite photocatalyst with interfacial chemical bonds was successfully prepared.

**Figure 2:**
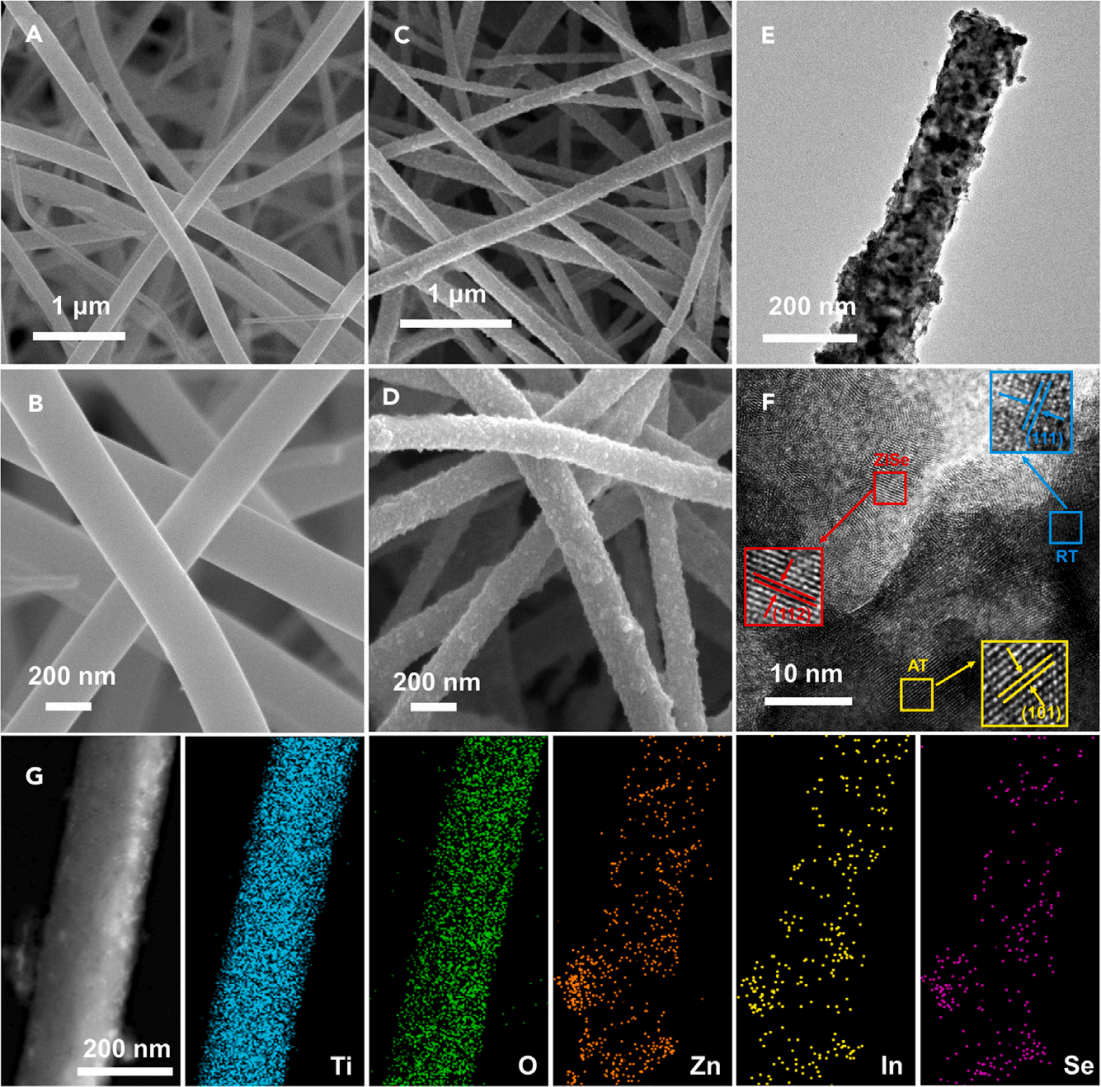
Morphology and composition characterizations (A and B) SEM of ART NFs. (C and D) SEM of ARTZ composite. (E and F) TEM of ARTZ composite. (G) EDS and elements mapping of Ti, O, Zn, In and Se.

The morphology and microstructure of the as-fabricated product ARTZ photocatalyst were further observed by scanning electron microscopy (SEM) and transmission electron microscopy (TEM). As shown in Figures 2A and 2B, the morphology of ART NFs with excellent length-diameter ratio was clearly displayed. The SEM images of ART NFs showed the characteristics of a smooth surface and uniform diameter, which provided a great environment for loading nanoparticles because of the large specific surface area. As exhibited in the Figures S3A–S3E, the clear visibility of the small ZISe NPs could be seen definitely and the elements in the field of view were evenly distributed. Besides, in Figures 2C and 2D, the ZISe NPs were uniformly and dispersively attached *in-situ* to the ART NFs. The microstructure of nanofibers loaded nanoparticles could be still precisely observed in the TEM image of Figure 2E. Figures S3F and S4A–S4E were analyzed using high resolution TEM (HRTEM). And the interface of ZISe NPs was in good contact with RT NFs as observed in Figure S4A. The effective contact between the nanofibers and the nanoparticles enhanced not only the active site of the reaction but also facilitated the separation and transportation of electric charge, resulting in improved photocatalytic solar energy conversion. The HRTEM of the composite ARTZ also confirmed the presence of AT, RT and ZISe in Figure 2F. The d-spacing of 0.341, 0.216 and 0.318 nm were assigned to the (101) lattice planes of AT, the (111) lattice planes of RT, and the (112) lattice planes of ZISe, respectively. As displayed in the Figure 2G, the images of elemental mapping present that Ti, O, Zn, In, and Se coexisted and were well-dispersed in the composite, further supporting the successful formation of ARTZ photocatalyst. The energy dispersive X-ray spectroscopy (EDX) of ARTZ is in Table S1. All the aforementioned characterizations prove that the composite ARTZ with ideal morphology structure and more active sites was synthesized and contributed to the improvement of activities.

### Photogenerated carriers mechanism

**Figure 3:**
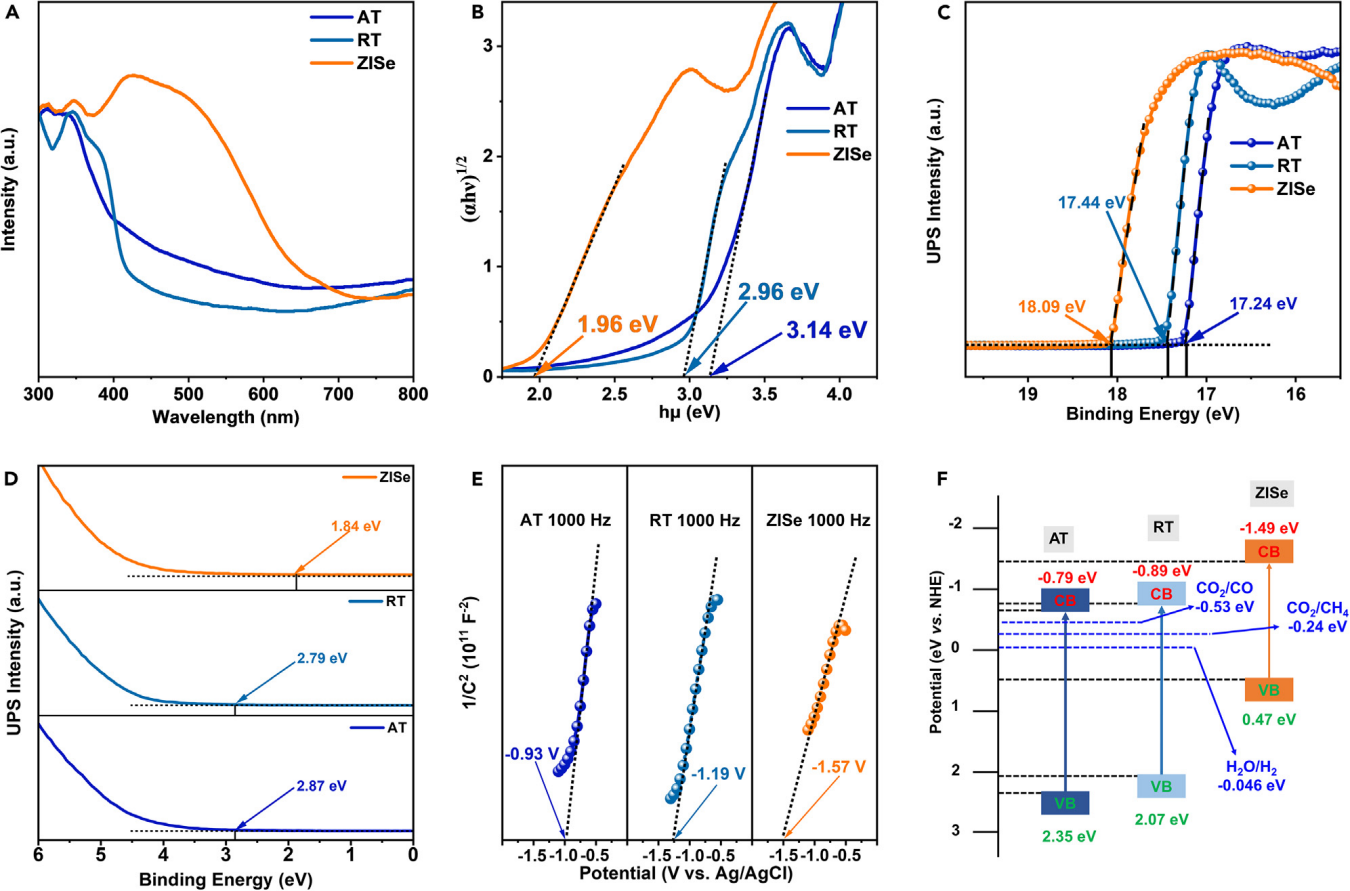
The band structure of samples (A) UV-vis absorption spectrum. (B) Kubelka-Munk function vs. the energy of incident light plots. (C and D) UPS spectra. (E) the Mott-Schottky plots. (F) the band alignment of AT, RT and ZISe.

The utilization of ZISe NPs with strong light absorption capacity (λ > 600 nm, Figure 3A) also enhanced the ability to harvest the sunlight of the composite. At the same time, the bandgap value (E_g_) of AT, RT, and ZISe could be obtained from the Kubelka-Munk function (αhν)^1/2^ and the energy of incident light plots (hν). As displayed in Figure 3B, the E_g_ of AT, RT, and ZISe were estimated to be 3.14, 2.96, and 1.96 eV, respectively. ZISe semiconductor NPs with narrower E_g_ in Figure 3B, proved that they possessed excellent sunlight capture, resulting in improving the efficiency of solar energy utilization in composite photocatalysts. Besides, we could investigate the photocatalytic mechanism of ARTZ by means of the ultraviolet photoelectron spectroscopy (UPS) with He I carried with an excitation energy of 21.22 eV in Figures 3C–3F. According to the Figure 3C, the cut-off edge energy (E_cut_) of as-prepared samples was calculated. While the work function (W_f_) could be further obtained from the following formula.

W_f_ = 21.22 - E_cut_.

Therefore, the W_f_ of AT, RT, and ZISe are 3.98, 3.78 and 3.13 eV vs. Vac. The Fermi level of three sample is −0.52, −0.72 and −1.37 eV. Next by the Figure 3D, the corresponding Fermi edge (E_f_) AT, RT and ZISe are 2.87, 2.79 and 1.84 eV, respectively. After then, the valence band potential (E_vb_) and the conduction band potential (E_cb_) also can be calculated by the following formulas (vs. Vac),

E_vb_ = - W_f_ - E_f_,

E_cb_ = E_vb_ + E_g_.

So the E_vb_ (vs. Vac.) of AT, RT, and ZISe were calculated. And the E_vb_ (vs. NHE) of as-prepared samples was equal to 2.35, 2.07 and 0.47 eV. Likewise, the values of E_cb_ (vs. NHE) were calculated, equivalent to −0.79, −0.72, and −1.49 eV, respectively. Nevertheless, the valence band potential of ZISe was too low to finish the complete redox reaction rapidly. The establishment of a double Z-scheme formed with AT and RT could work this challenge out. Combined with the flat band potential of AT, RT, and ZISe in Figure 3E, it could be confirmed that the above materials were n-type semiconductors and the data derived from the UPS test was reliable. All the above values for the energy bands are exhibited in Table S2. From the above testing results and analyzing data, the energy band structure of AT, RT, and ZISe with ideal energy band matching among three semiconductors was presented in Figure 3F. The energy band position of ZISe could be observed to indicate that the conduction band position of ZISe is at a more negative position, which suggests that it possesses excellent photogenerated electron reduction characteristics. The Figure 3F illustrates that the conduction position and Fermi level of RT are more negative than those of AT. Consequently, when the two semiconductors form an intimate interface contact, the free electrons of RT spontaneously migrate to AT through the interface. Simultaneously, the Fermi level was flattened and the energy band was bending, resulting in RT bending upwards and AT bending downwards, thereby creating an internal electric field between AT and RT.

**Figure 4:**
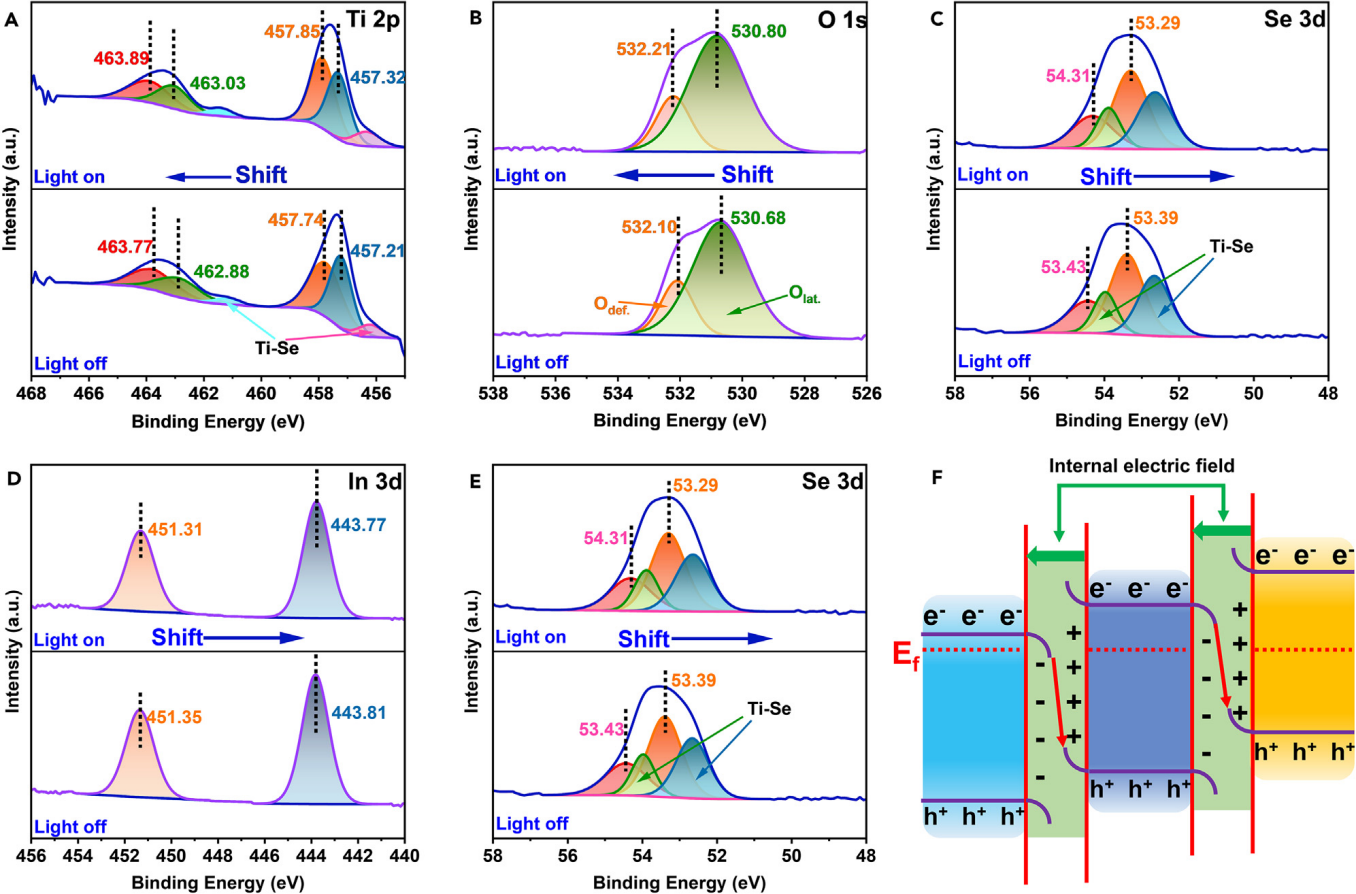
ISI-XPS spectra of ARTZ (A) Ti 2p, (B) O 1s, (C) Zn 2p, (D) In 3d and (E) Se 3d for ARTZ composite, (F) the photogenerated charges transfer pathways of ARTZ.

In order to distinguish the transfer direction of electrons in the system more intuitively and expressly, the *in situ* irradiated X-ray photoelectron spectroscopy (ISI-XPS) was adopted.^56^^,^^57^^,^^58^ The analysis principle of ISI-XPS was shown in the Figure S5A and the changes of binding energy were tested during the photocatalytic process by ISI-XPS in Figures 4A–4E. To simulate the environment in which the photocatalytic reaction occurs, an external light source can be added during the test process. This allows for the direct estimation of the transfer direction of photogenerated electrons through the movement of characteristic peaks. As observed in Figures 4A and 4B, the ISI-XPS peaks of Ti and O obviously shifted toward a higher position during the light irradiation, indicating that the photogenerated electrons flowed from the ART NFs side. Additionally, under light irradiation, the binding energy of Zn 2p, In 3 days and Se 3 days of ARTZ showed a negative shift, suggesting that ZISe NPs acted as the receiver of photogenerated charges. The energy band position of AT and RT has been obtained, resulting in the photogenerated electrons migration from the conduction position to the valence position. Besides, compared with AT, the RT containing the OVs is more likely to attract and trap photogenerated electrons, noting that electrons tended to flow toward the RT side in a system of ART. Herein, this conclusion could be drawn that the direction of photogenerated electrons flowed from AT to RT, then to ZISe, forming a double Z-Scheme heterojunction photocatalyst. The XPS results verified the successful synthesis of the compound with an interfacial Ti-Se bond ulteriorly. The interfacial chemical bonds as transport channel facilitated the transmission of electrons from RT to ZISe. Additionally, the strong combination of AT, RT, and ZISe created the internal electric fields at the interface of these three semiconductors (Figures S5B–S5D, as well as Figure 4F). The construction of internal electric fields provided a driving force for the electrons-directed transport, which promoted the fast occurrence of the reaction and the major improvement of the performance.

### Photoelectrochemical characterization and photocatalytic performance

**Figure 5:**
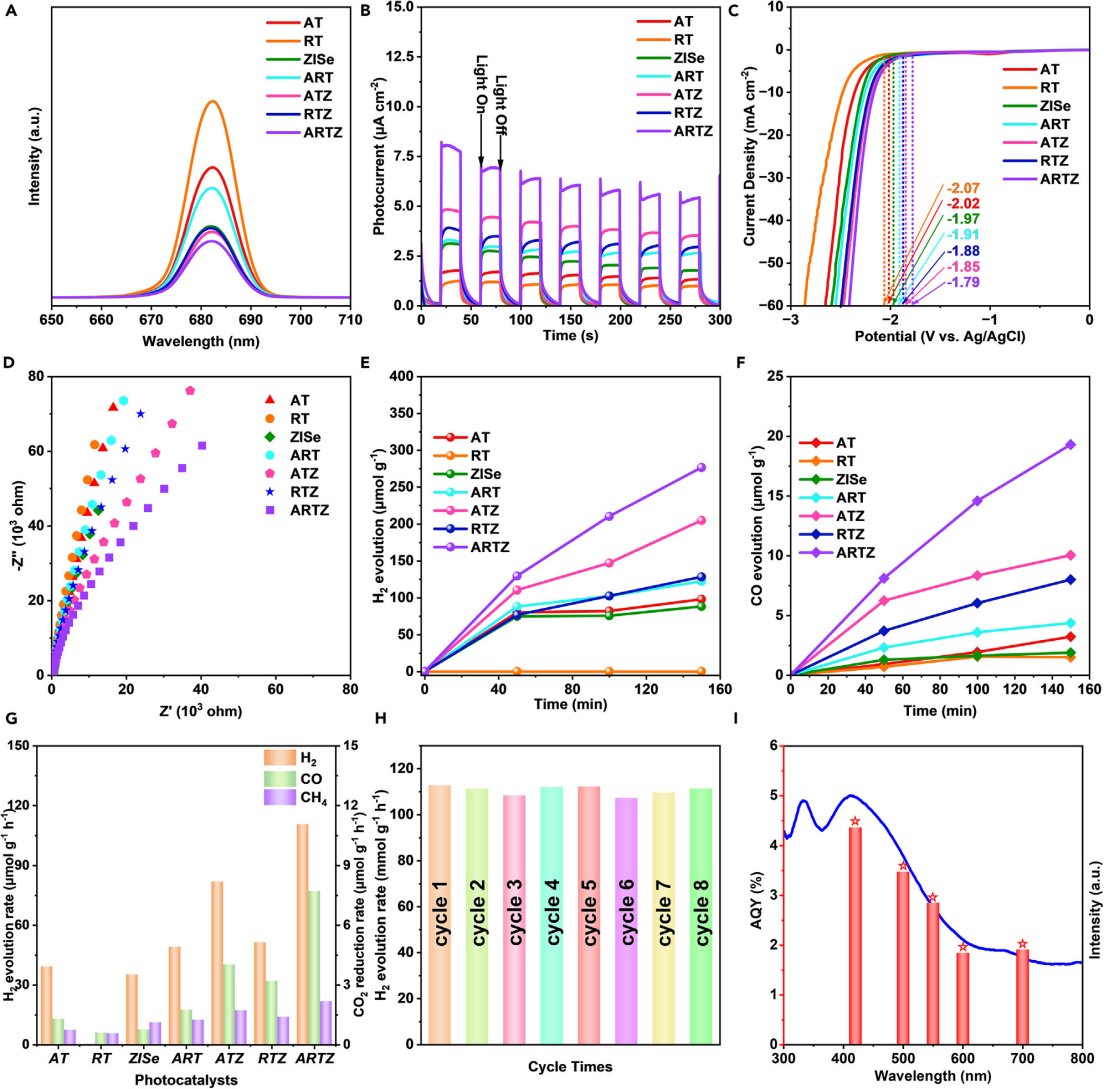
Photoelectrochemical characterization and photocatalytic performance (A) the photoluminescence (PL). (B) the photocurrent response (i-t). (C) the linear sweep voltammogram (LSV). (D) the electrochemical impedance (EIS). (E) H_2_ evolution. (F) CO evolution. (G) H_2_, CO and CH_4_ evolution rate of samples. (H) cycling stability test of ARTZ. (I) wavelength-dependent apparent quantum yield (AQY) of ARTZ.

The photoelectrochemical tests in Figures 5A–5D could further verify the optimized charge separation and transfer efficiency of ARTZ composition. In Figure 5A of PL spectroscopy, the photogenerated carrier recombination of ZISe NPs was weaker than AT and RT under the light irradiation, illustrating the stronger absorption and utilization of light. The ARTZ composite exhibited a minimum emission peak, indicating lower photocarrier recombination efficiency and higher separation efficiency. Compared to AT and RT, the photocurrent density, the linear sweep voltammogram (LSV), and the electrochemical impedance (EIS) of ART were more excellent (Figures 5B–5D). This was due to the tightly integrated hetero-phase interface and the band structure of ART homojunction, which weakened the self-recombination of the photogenerated carriers and improved the transfer efficiency of interfacial charge. Although the photoelectric performance of ATZ and RTZ was promoted compared to original AT, RT, and ZISe, they were still at a slight disadvantage compared to ARTZ composite. This is due to the inhibiting influence of homojunction on photocarrier recombination, the driving force of the internal electric field on charge transmission, and the channel effect of interfacial chemical bonds on electron transfer. The combined impact of these three factors significantly enhanced the photoelectric performance. The double Z-scheme ARTZ composite photocatalyst exhibited extremely high photocurrent density, outstanding LSV, and depressed EIS. These results were attributed to the efficient separation and transmission of photogenerated carriers, which demonstrated the superior photochemical properties of the composite photocatalyst. The precise separation and transfer of photogenerated electrons and holes further improved the photocatalytic efficiency. Thus, the double Z-scheme heterojunction of ARTZ photocatalyst with perfect energy band matching is an unparalleled design.

To evaluate the photocatalytic solar energy conversion, we compared the the hydrogen evolution rate (HER) and CO_2_ reduction rate of samples. As shown in Figures 5E and 5F, the photocatalytic performance of all samples for H_2_ evolution and CO evolution is exhibited and compared. The evolution curves demonstrated an upward trend, and the performance improvement of the ARTZ sample was the most obvious among them. The H_2_ evolution of ZISe, ART, and ARTZ was 0.088, 0.097 and 0.277 mmol g^−1^, respectively. Similar results were obtained in the tests of photocatalytic CH_4_ evolution (Figure S6). For the photocatalytic cycling tests with ARTZ composite, which exhibited the best performance, was selected as the representative sample. As shown in Figure 5G, the H_2_, CO, and CH_4_ evolution rate was detected. The HER of ARTZ was up to 0.11 mmol g^−1^ h^−1^, which was 3.15 times of the original ZISe NPs. The CO_2_ reduction measurement program primarily detected the photocatalytic index of CO and CH_4_ evolution. The ARTZ compound still exhibited the best activity indicators, with 7.72 μmol g^−1^ h^−1^ of CO evolution and 2.18 μmol g^−1^ h^−1^ of CH_4_ evolution. The rapid transport and exchange of photogenerated carriers was due to the connection of interfacial chemical bonds and the presence of the internal electric field. As a result, the reaction at the interface was faster, leading to a significant improvement in the hydrogen production rate.

The results were conducted under the same conditions to evaluate the cycling stability, as shown in Figure 5H. After eight cycles of 20 h, the hydrogen evolution rate remained still at a similar height without dropping, revealing that the ARTZ photocatalyst exhibited excellent cycling stability and remarkable photocatalytic activity. Also in Figure S7, the peaks of the XPS pattern in the ARTZ composite photocatalyst after testing exhibited minimal shift, indicating that the structure of the ARTZ composite remained unaltered and exhibited excellent stability. The ARTZ was conducted for the photocatalytic cycling test and apparent quantum yield (AQY) of the photon utilization efficiency in Figure 5I, which achieved 4.36% at 420 nm. This double Z-scheme heterostructure reduced the complexation of photogenerated electrons and holes, facilitated charge separation and migration, and exposed more active sites. This enhanced the adsorption and catalysis of CO_2_. The results of the photocatalytic performance test, as described above, illustrated that the as-prepared composite photocatalysts prepared in this study significantly boosted both the photocatalytic activity and chemical stability.

### Photocatalytic mechanisms of solar energy conversion

**Figure 6:**
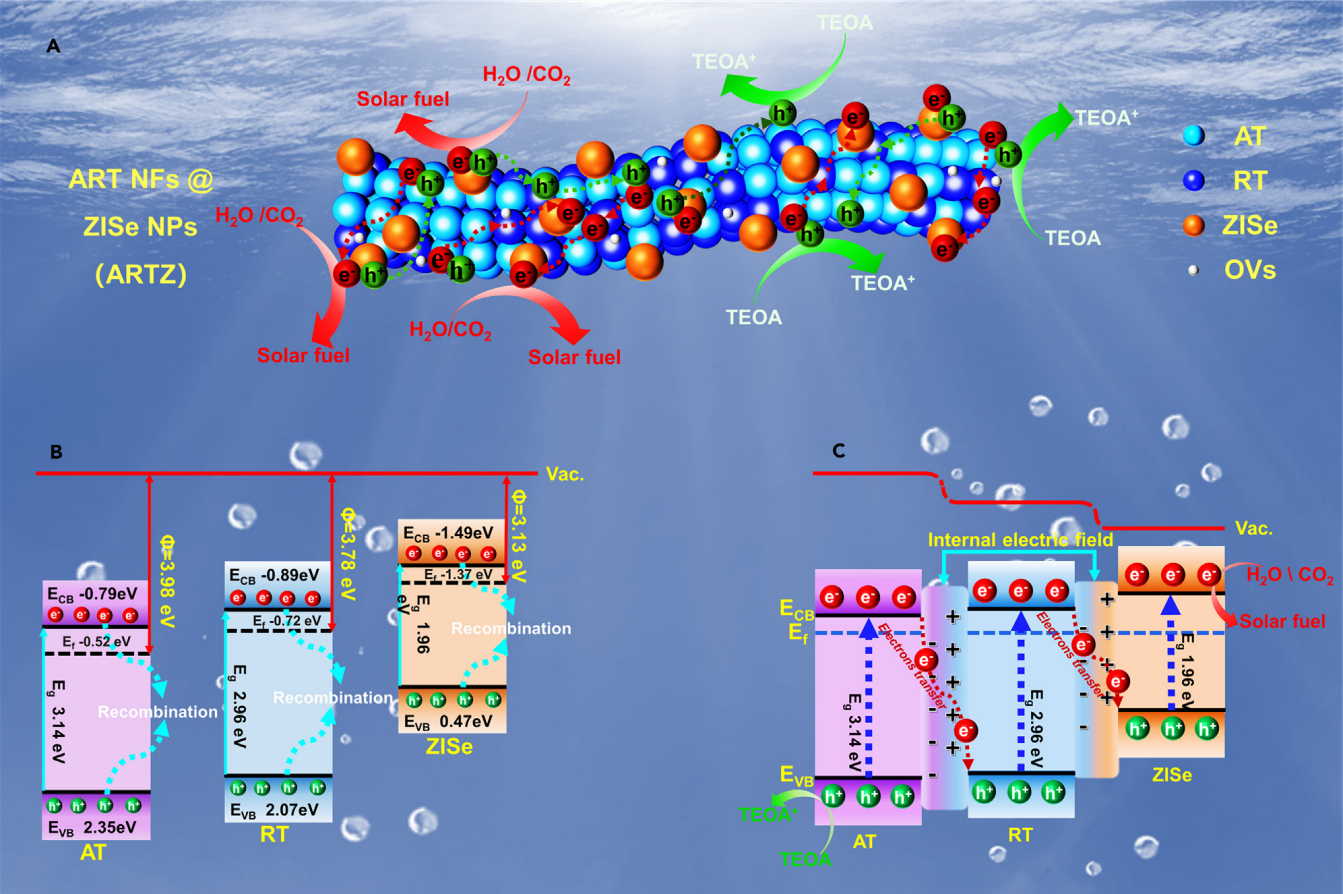
The Mechanism of ARTZ (A) the schematic illustration of charge transfer pathways in AT, RT and ZISe. (B) the band structure of AT, RT and ZISe. (C) the photogenerated charges transfer pathways in ARTZ.

Based on the analysis of the above experimental data results, the mechanism transmission of photogenerated electrons and holes among ZISe, RT, and AT could be drawn in the Figure 6. The photocatalysts were connected through heterophase homojunction, internal electric fields, and interfacial chemical carriers. The problem, the severe recombination of photogenerated carriers and the small number of active sites, could be addressed effectively by constructing a double Z-scheme heterojunction which was composed of one-dimensional TiO_2_ nanofibers and ZnIn_2_Se_4_ nanoparticles. As illustrated in Figure 6B, the energy band structure of AT, RT, and ZISe exhibited a gradient distribution before contact. In Figure 6C, the system is susceptible to degradation when exposed to sunlight. The electrons in the valence band of AT were excited and jumped to the conduction band after absorbing sunlight, and then rapidly transported to the valence band of RT under the driving force of the internal electric field, where they were recombinated with the excited photogenerated holes. At the same time, the photogenerated electrons in the conduction band of RT were rapidly transported to the valence band of ZISe under the double action of the internal electric field and the interfacial chemical bond. The excited photogenerated charge remained in the conduction band and participated in the reduction reaction.

The double Z-scheme charge transport mechanism significantly enhanced the effective separation of electrons and holes. Firstly, the construction of the composite photocatalyst comprising ART NFs and ZISe NPs effectively solves the problem of nanoparticle agglomeration and carrier recombination, thereby increasing the number of reactive sites and facilitating the separation and migration of photogenerated carriers. Secondly, the formation of the internal electric field and the interfacial chemical bonding after the contact, with the former providing a driving force for the transport of photogenerated carriers and the latter acting as a fast channel for electron transport, both of which are conducive to the migration of electrons and the promotion of the photocatalytic reaction. In summary, this work presented a double Z-scheme heterojunction constructed utilizing the mixed-phase TiO_2_ and the novel bimetallic selenide ZnIn_2_Se_4_. This approach effectively boosted the separation and transmission of carriers, thereby promoting the efficiency of photocatalytic solar fuel preparation.

### Conclusion

In conclusion, a heterojunction composite photocatalyst of ART NFs and ZISe NPs with a double Z-scheme heterojunction was successfully prepared through the simple electrospinning technology and solvothermal method. Chalcopyrite ZISe NPs, which exhibited outstanding light absorption, were synthesized for use in the photocatalytic preparation of solar fuel. By utilizing mixed-phase TiO_2_ NFs, the aggregated ZISe NPs were dispersed, increasing the number of active sites for the reaction and resulting in the improved photocatalytic activity. And the reduction reaction occurs in the conduction band of ZISe, which exploits the reducing nature of its photogenerated electrons to the fullest extent and facilitates the photocatalytic reaction. Additionally, the internal electric fields and interfacial chemical bonds in the double Z-scheme heterojunction system provided the driving force for the directional transmission of electrons and established the transport channel for the efficient transfer of charge. The synergistic action of these factors greatly increased the efficiency of photocatalytic solar fuel preparation. Herein, the formation of ZISe photocatalysts with a particular chalcopyrite structure based on solvothermal technology significantly provides an innovative opinion for the development of future photocatalysts.

### Limitations of the study

We constructed a double Z-scheme heterojunction of ZnIn_2_Se_4_ semiconductor and confirmed its photocatalytic activity. Although this material has been confirmed to have the photocatalytic performance of preparing solar fuel, it still has certain limitations in photocatalysis. ZnIn_2_Se_4_ nanoparticles prepared in this study have no fixed shape and uneven size, and it is difficult to obtain an ideal morphology, which may be one of the reasons for restricting their photocatalytic activity.

## STAR★Methods

### Key resources table

**Table:** 

REAGENT or RESOURCE	SOURCE	IDENTIFIER
**Antibodies**		

Polyvinylpyrrolidone	Macklin	9003-39-8
Ethanol absolute	Sinopharm Chemical Reagent Co., Ltd	64-17-5
Glacial acetic acid	Sinopharm Chemical Reagent Co., Ltd	64-19-7
Titanium butoxide (TBOT, ≥99.0%)	aladdin	5593-70-4
Hydrazine monohydrate (N2H4·H2O, AR)	kermel	7803-57-8
Zinc acetate (AR)	Macklin	557-34-6
Indium nitrate hydrate (≥ 99.5%)	Sinopharm Chemical Reagent Co., Ltd	207398-97-8
Selenium (99.9% metal basis)	Macklin	7746-08-4
Triethanolamine (TEOA, AR)	Sinopharm Chemical Reagent Co., Ltd	102-71-6

### Resource availability

#### Lead contact

Further information and requests for resources and reagents should be directed to and will be fulfilled by the lead contact, Peng Zhang(zhangp@zzu.edu.cn).

#### Materials availability

This study did not generate new unique reagents.

#### Data and code availability

All original data in this paper will be shared by the lead contact upon request.

Any additional information required to reanalyze the data reported in this paper is available from the lead contact upon request.

This paper does not cover original code.

### Experimental model and study participant details

This paper does not cover the content of experimental model and study participant details.

### Method details

#### Preparation of TiO_2_ nanofibers

First, 1.1 g of PVP powder was dissolved in a mixture solution of 10 ml of Ethanol absolute and 6 ml of Glacial acetic acid for 12 hours of stirring at room temperature. Then, 2 ml of Titanium butoxide was added to the above solution and keep stirring for two hours. After then, all of the mixed system was dumped into a syringe to start spinning. And the instrument used in the further process is electrospinning machine (UCALERY, ET-3556H), at the positive voltage power of 15 kV and negative voltage power of -5 kV. In the process of electrospinning, the distance between the syringe needle and the receiving port was set as 20 cm. Waiting for electrospinning to finish, the white spinning film was removed and calcined in a Muffle furnace. At 450, 600 and 800°C for 2h with a heating rate of 5°C/min in the atmosphere, the white products turned to anatase, mixed-phase and rutile TiO_2_ nanofibers, respectively. We labeled these compounds as AT, ART and RT, severally.

#### Fabrication of the composite

The composite was prepared via the in-situ solvothermal method. A certain amount of (CH_3_COO)_2_Zn and In(NO_3_)_3_·4.5H_2_O were dissolved in 15 ml of Ethanol absolute, and the selenium powder was added to 10 ml of N_2_H_4_·H_2_O. After stirring separately at room temperature for half an hour, mix together and stir for another twelve hours. When prefabricating the solution, the mole ratio was 1:1:4 of Zn, In and Se. Then pure ZnIn_2_Se_4_ production powder was synthesized at 180°C for 12h by solvothermal method, labeled as ZISe NPs. Similarly, the ATZ, RTZ and ARTZ composite was prepared through adding AT, RT and ART NFs to the mixed solvent by in-situ solvothermal, respectively. The mass ratio of TiO_2_ NFs and ZISe NPs was 1:3.

#### Characterizations

Using a Rigaku Ultima IV system with Cu Kα radiation (λ = 1.54056 Å) at 50 kV of voltage and 200 mA of current in the 2θ range of 10 to 90° at a scanning rate of 20°·min^-1^, the powder X-ray diffraction (XRD) was performed to identify the crystalline structure and phase composition. The scanning electron microscopy (SEM, ZEISS SIGMA-500) and the transmission electron microscopy (TEM, FEI Tecnai G20) was convenient to detect the micro-morphology and lattice fringes at the interface of sample. The X-ray photoelectron spectroscopy (XPS), ultraviolet photoelectron spectroscopy (UPS) and in suit irradiation X-ray photoelectron spectroscopy (ISI-XPS) were utilized to study the energy band matching and electrons transport direction. Thereof the Ultraviolet-visible diffuse reflectance spectrometer (UV-Vis DRS, UV-3600, SHIMADZU) used BaSO_4_ powder as a standard quantification to detect. The electrochemical workstation (CHI760E) was used to test the photocurrent response (i-t), electrochemical impedance spectroscopy (EIS), linear sweep voltammetry (LSV) and Mott-Schottky (MS) experiments.

#### Photocatalytic H_2_ evolution

In the photocatalytic H_2_ evolution experiment, samples were dispersed in a mixed solution containing 50 ml of deionized water and 10 ml of TEOA. Then, the gas chromatograph (GC-2014, SHIMADZU) was carried out to analyse 16 ml of mixed gas with 1 ml of H_2_ produced by samples and 15 ml of argon. During the cycling test, the sample would be reused 4 times for a total of 600 minutes to proceed the same photocatalytic H_2_ evolution. As for apparent quantum yield (AQY) of photocatalyst, there would be tested at different wavelength of light with the help of 420, 500, 550, 600, 700 nm bandpass filters. The results were dealt with the following formula,

AQY = N_e_/N_p_ × 100% = (2 × n_H2_ × h × c) / (P × t × λ) × 100%.

In which, the N_e_ and N_p_ represent the number of reacted electrons and incident photons, respectively. The n_H2_ refers to the number of evolved H_2_ molecules, while the h and c mean the Planck constant (6.63×10^-34^ J·s) and light speed (3.0×10^8^ m·s^-1^), respectively. The light power is denoted as P, and the illumination time is referred to t. The λ denotes the wavelength of incident light.

#### Photocatalytic CO_2_ reduction

In this test program, 20 ml of CO2 was propelled in the reactor before the test. Then, the photocatalytic CO2 reduction measurement was performed as the same technological process like photocatalytic H2 evolution test.

### Quantification and statistical analysis

This paper does not cover the content of quantitative and statistical analysis.
