# Supplementary material for: ZnIn2Se4 nanoparticles photocatalyst for efficient solar fuel production
Source: iScience. 2024 Jun 29;27(8):110422. doi: 10.1016/j.isci.2024.110422 (PMC11301066; doi:10.1016/j.isci.2024.110422)
Supplement: Document S1. Figures S1–S7 and Tables S1 and S2 [file mmc1.pdf]

iScience, Volume *27*

Supplemental information

$ZnIn_2Se_4$ nanoparticles photocatalyst for efficient solar fuel production

Yinyin Ai, Yukun Li, Ting Li, Ruohan Hou, Qing Wang, Aneela Habib, Guosheng Shao, and Peng Zhang

Supplemental information

$ZnIn_2Se_4$ nanoparticles photocatalyst for efficient solar fuel production

Yinyin Ai, Yukun Li, Ting Li, Ruohan Hou, Qing Wang, Aneela Habib, Guosheng Shao and Peng Zhang

Contents

Figure S1. The details of synthesis method and crystal structure of photocatalyst. Related to Figure 1. (a) the preparation of photocatalysts, (b) the crystal structure of AT, RT and ZISe.

Figure S2. Comparison of XPS diffraction peaks spectra between ZISe and composite photocatalyst. Related to Figure 1. (a) The XPS spectra of Zn 2p, (b) the XPS spectra of In 3d for ZISe and ARTZ.

Figure S3. The morphology of ZISe and composite photocatalyst. Related to Figure 2. (a-b) The SEM image of $ZnIn_2Se_4$ NPs, (c-e) the EDS and elements mapping of Zn, In and Se, (f) the TEM image of ARTZ.

Figure S4. Lattice fringe diagram of composite photocatalyst ARTZ. Related to Figure 2. (a-b) the TEM image of ARTZ, (c) the HRTEM, IFFT and profile of IFFT images of AT, (d) the HRTEM, IFFT and profile of IFFT images of RT, (e) the HRTEM, IFFT and profile of IFFT images of ZISe.

Figure S5. Diagram of photocatalytic mechanism analysis with ISI-XPS. Related to Figure 4. (a) The mechanism of ISI-XPS, (b) the band structure of AT, RT and ZISe, (c) the establishment of internal electric field among AT, RT and ZISe, (d) electrons transfer mechanism of ARTZ composite under the light.

Figure S6. Figure S6. The photocatalytic CH_4 evolution test over samples. Related to Figure 5.

Figure S7. XPS spectra of composite after test. Related to Figure 5. (a) ARTZ, (b) Ti 2p, (c) O 1s, (d) Zn 2p, (e) In 3d and (f) Se 3d for ARTZ.

Table S1. The energy dispersive X-ray spectroscopy (EDX) of ARTZ. Related to Figure 2.

Table S2. The potential of AT, RT and ZISe. Related to Figure 3.

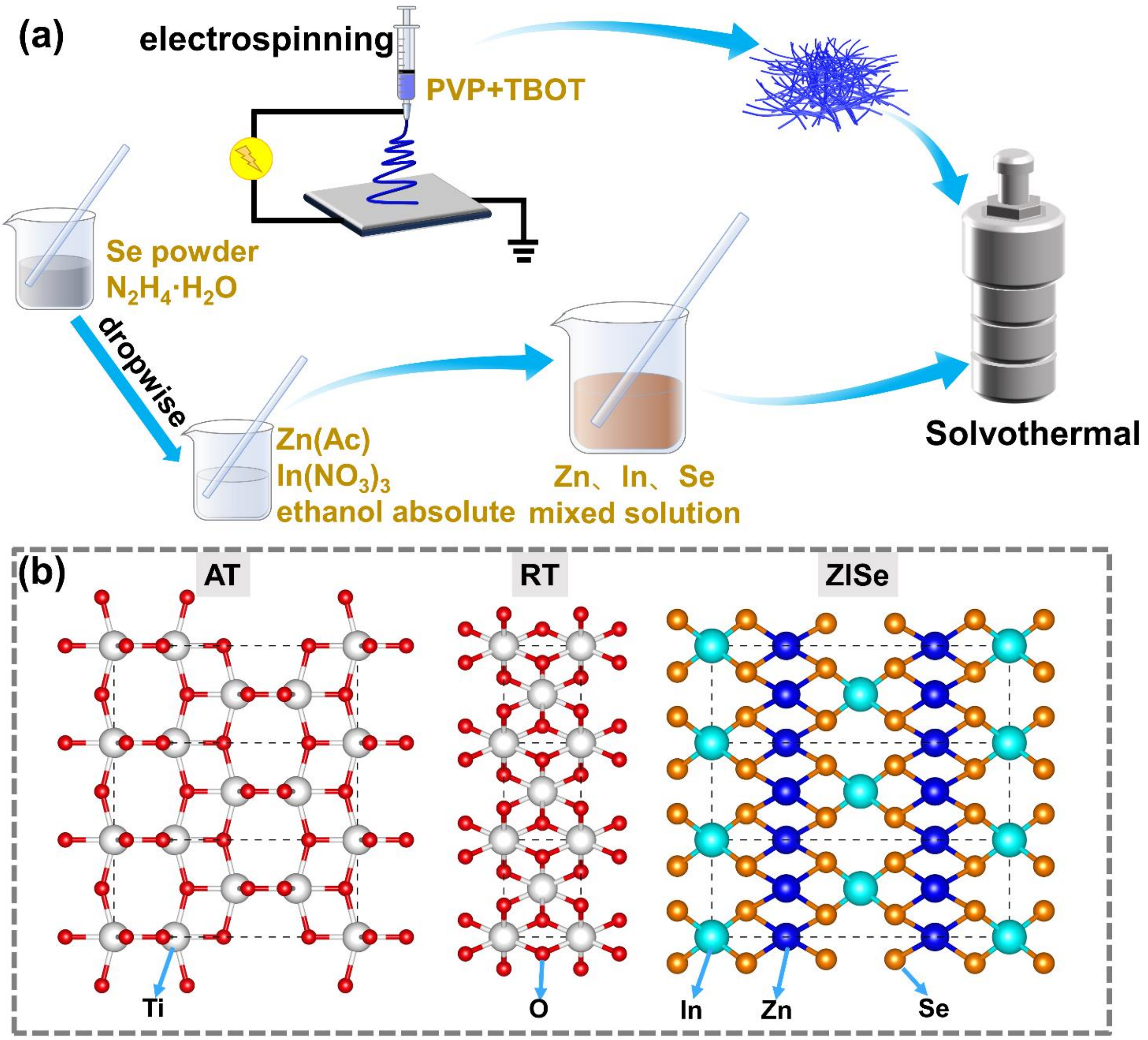

Figure S1. The details of synthesis method and crystal structure of photocatalyst. Related to Figure 1. (a) the preparation of photocatalysts, (b) the crystal structure of AT, RT and ZISe.

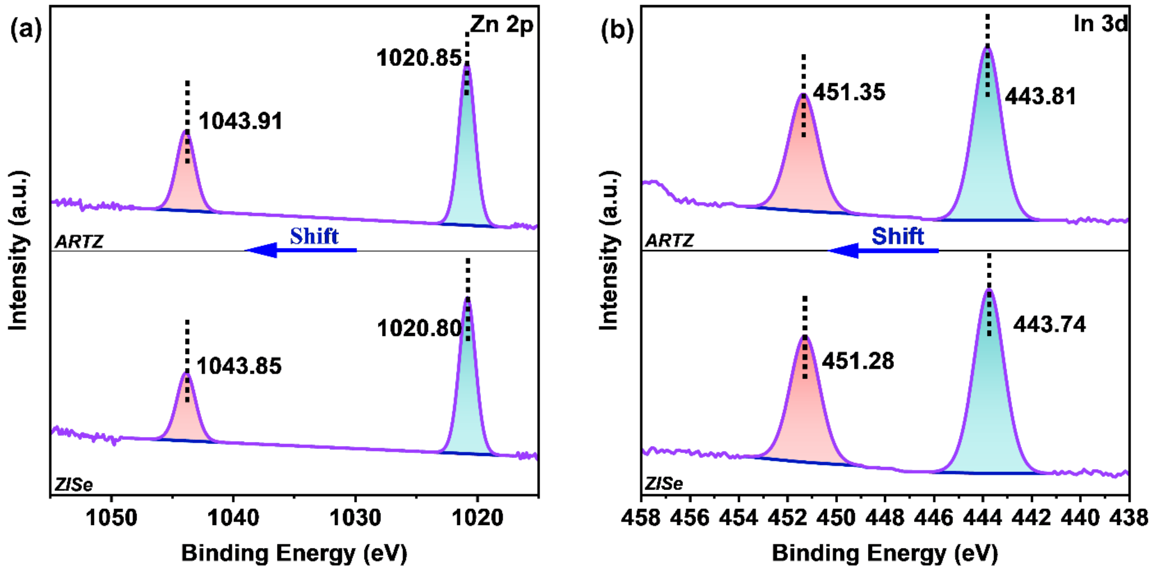

Figure S2. Comparison of XPS diffraction peaks spectra between ZISe and composite photocatalyst. Related to Figure 1. (a) The XPS spectra of Zn 2p, (b) the XPS spectra of In 3d for ZISe and ARTZ.

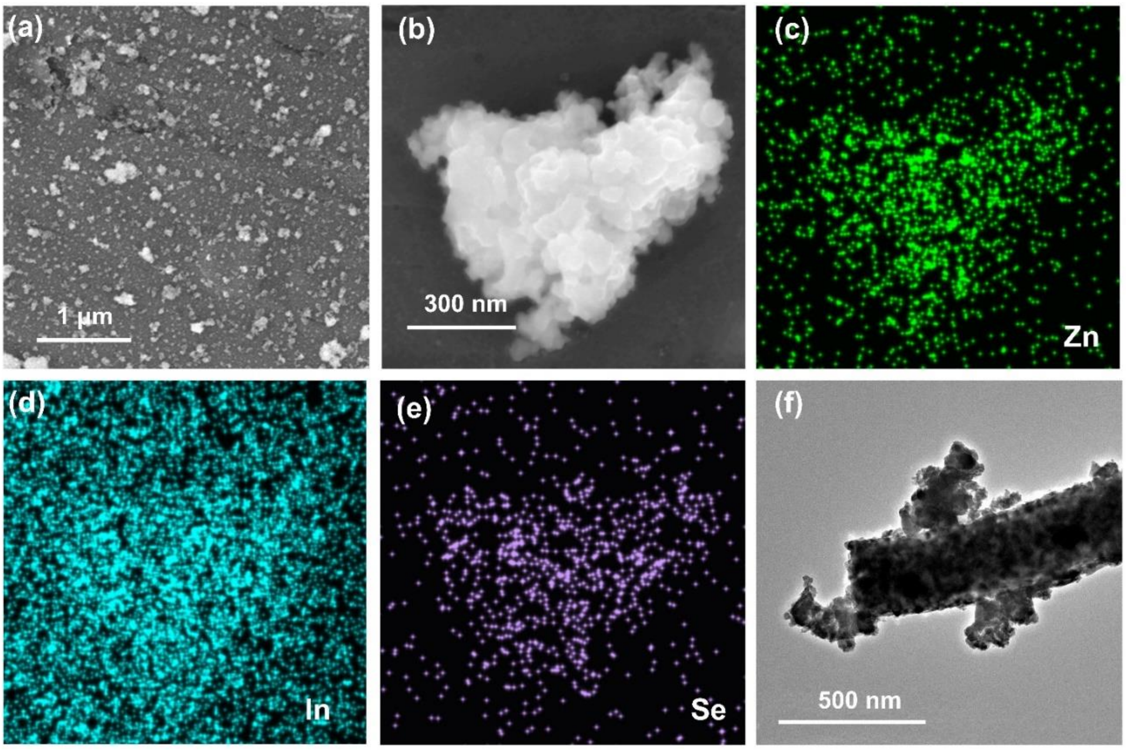

Figure S3. The morphology of ZISe and composite photocatalyst. Related to Figure 2. (a-b) The SEM image of $ZnIn_2Se_4$ NPs, (c-e) the EDS and elements mapping of Zn, In and Se, (f) the TEM image of ARTZ.

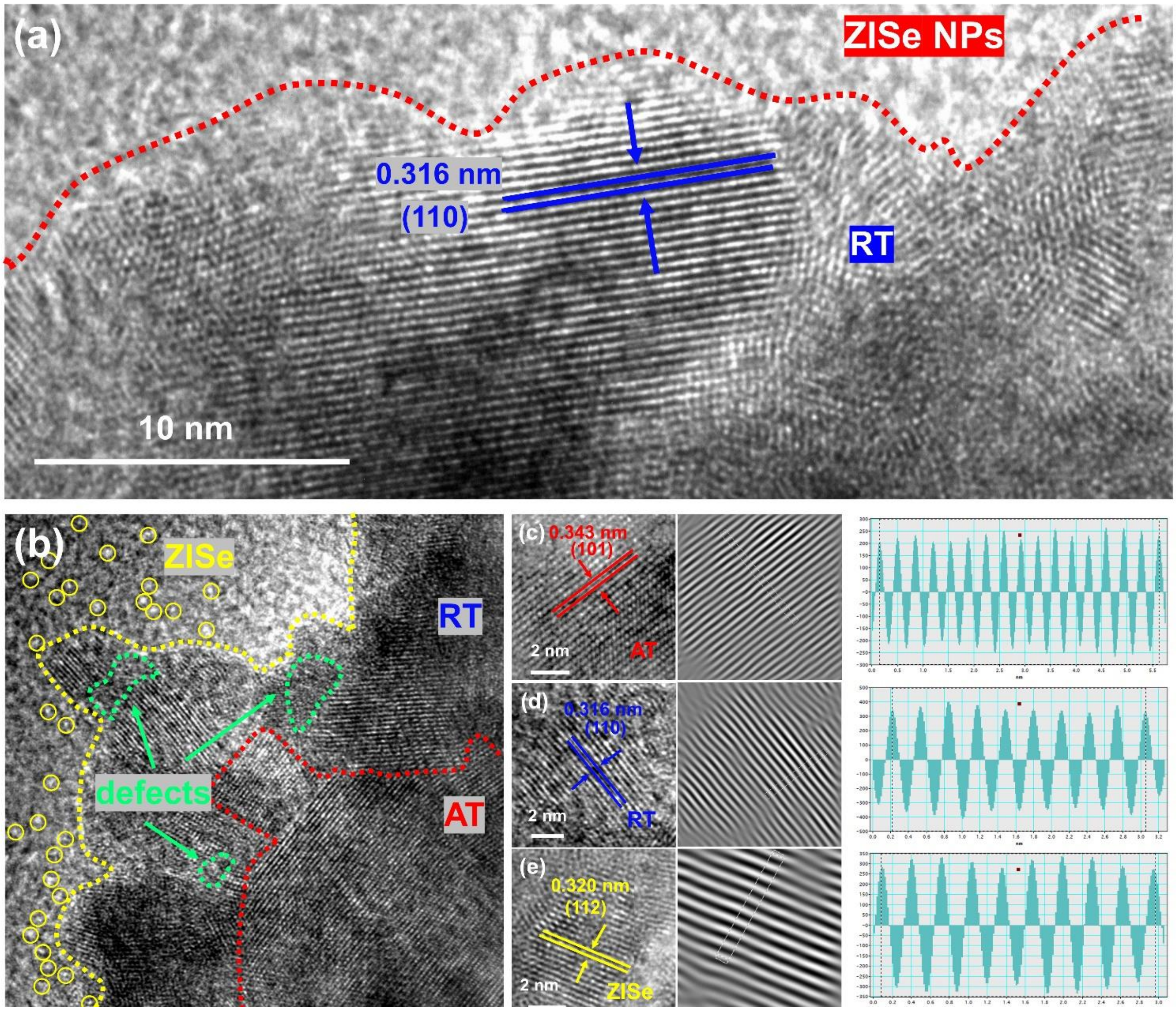

Figure S4. Lattice fringe diagram of composite photocatalyst ARTZ. Related to Figure 2. (a-b) the TEM image of ARTZ, (c) the HRTEM, IFFT and profile of IFFT images of AT, (d) the HRTEM, IFFT and profile of IFFT images of RT, (e) the HRTEM, IFFT and profile of IFFT images of ZISe.

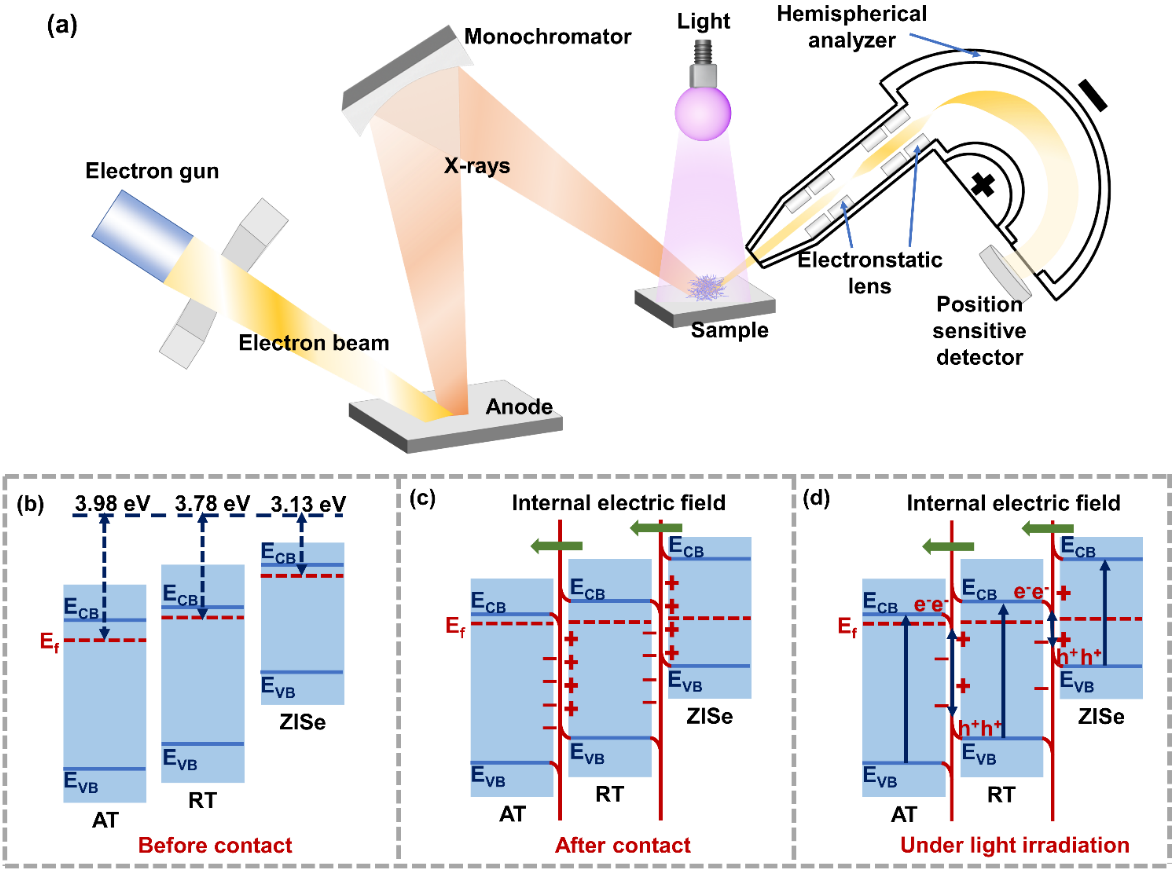

Figure S5. Diagram of photocatalytic mechanism analysis with ISI-XPS. Related to Figure 4. (a) The mechanism of ISI-XPS, (b) the band structure of AT, RT and ZISe, (c) the establishment of internal electric field among AT, RT and ZISe, (d) electrons transfer mechanism of ARTZ composite under the light.

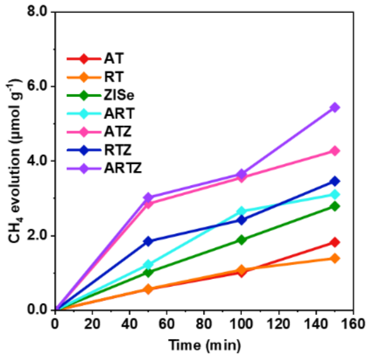

Figure S6. The photocatalytic CH_4 evolution test over samples. Related to Figure 5.

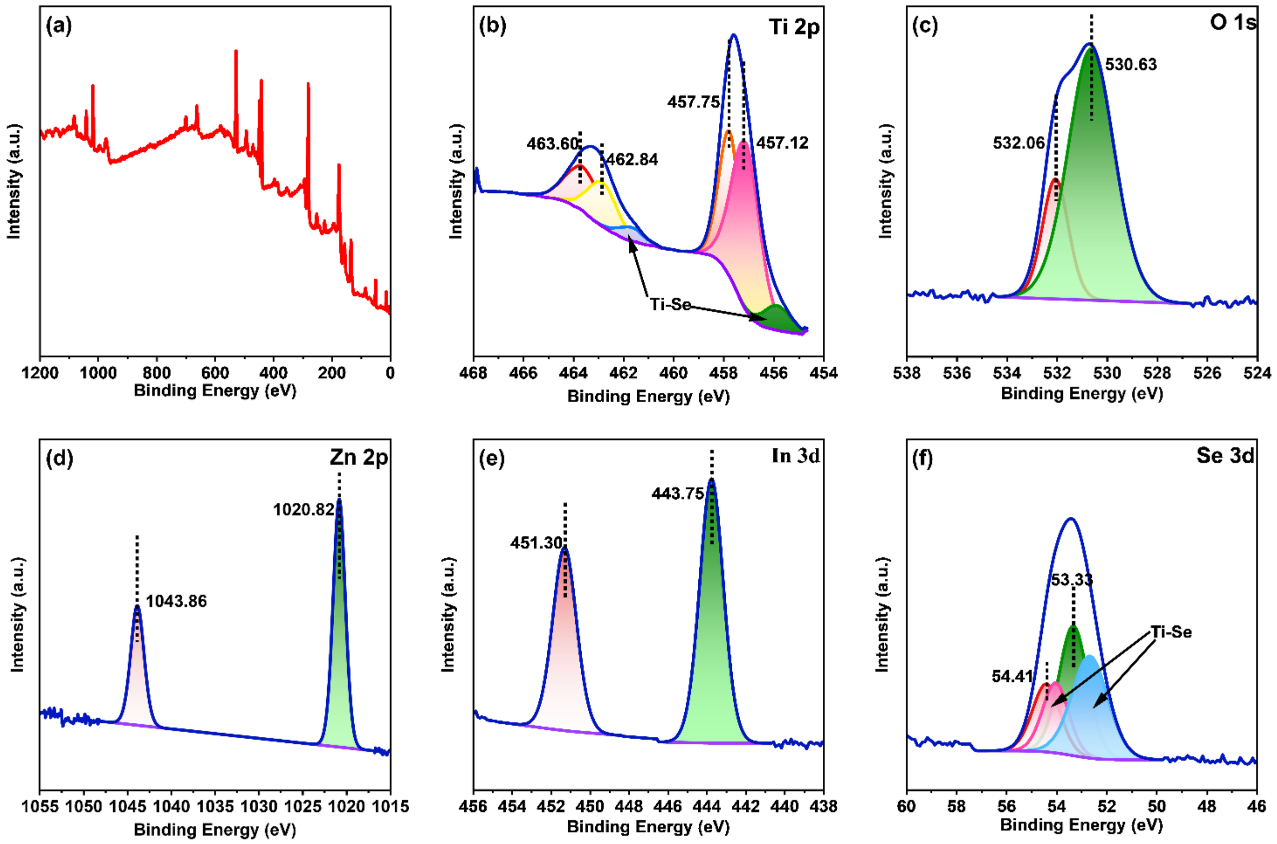

Figure S7. XPS spectra of composite after test. Related to Figure 5. (a) ARTZ, (b) Ti 2p, (c) O 1s, (d) Zn 2p, (e) In 3d and (f) Se 3d for ARTZ.

Table S1. The energy dispersive X-ray spectroscopy (EDX) of ARTZ. Related to Figure 2.

		ART NFs@ZISe NPs (ARTZ)		
elements	atomicity	normalized mass (%)	atom (%)	Abs.error (%) (3 sigma)
Ti	22	41.54	19.96	2.66
O	8	55.02	79.08	12.80
Zn	30	0.51	0.18	0.19
In	49	0.78	0.16	0.20
Se	34	2.15	0.63	0.31

Table S2. The potential of AT, RT and ZISe. Related to Figure 3.

			Potential of ART NFs@ZISe NPs (ARTZ) /eV		
samples	E_g	E_{cut}	Fermi level (vs. NHE)	E_{vb} (vs. NHE)	E_{cb} (vs. NHE)
AT	3.14	17.24	-0.52	2.35	-0.79
RT	2.96	17.44	-0.72	2.07	-0.89
ZISe	1.96	18.09	-1.37	0.47	-1.49
